# Accommodating error analysis in comparison and clustering of molecular fingerprints.

**DOI:** 10.3201/eid0402.980203

**Published:** 1998

**Authors:** H Salamon, M R Segal, A Ponce de Leon, P M Small

**Affiliations:** University of California, San Francisco, USA. hugh@molepi.stanford.edu

## Abstract

Molecular epidemiologic studies of infectious diseases rely on pathogen genotype comparisons, which usually yield patterns comprising sets of DNA fragments (DNA fingerprints). We use a highly developed genotyping system, IS6110-based restriction fragment length polymorphism analysis of Mycobacterium tuberculosis, to develop a computational method that automates comparison of large numbers of fingerprints. Because error in fragment length measurements is proportional to fragment length and is positively correlated for fragments within a lane, an align-and-count method that compensates for relative scaling of lanes reliably counts matching fragments between lanes. Results of a two-step method we developed to cluster identical fingerprints agree closely with 5 years of computer-assisted visual matching among 1,335 M. tuberculosis fingerprints. Fully documented and validated methods of automated comparison and clustering will greatly expand the scope of molecular epidemiology.


### Accommodating Error Analysis in Comparison and Clustering of Molecular Fingerprints

**Hugh Salamon,* Mark R. Segal,***
**Alfredo Ponce de Leon,† and Peter M. Small‡**
*University of California, San Francisco, California, USA;
†Instituto Nacional Nutrición, Zubriran, Mexico City, Mexico; and
‡Stanford University Medical Center, Stanford, California, USA

Molecular epidemiologic studies of infectious diseases rely on pathogen genotype comparisons, which usually yield patterns comprising sets of DNA fragments (DNA fingerprints). We use a highly developed genotyping system, IS*6110*-based restriction fragment length polymorphism analysis of *Mycobacterium tuberculosis*, to develop a computational method that automates comparison of large numbers of fingerprints. Because error in fragment length measurements is proportional to fragment length and is positively correlated for fragments within a lane, an align-and-count method that compensates for relative scaling of lanes reliably counts matching fragments between lanes. Results of a two-step method we developed to cluster identical fingerprints agree closely with 5 years of computer-assisted visual matching among 1,335 *M. tuberculosis* fingerprints. Fully documented and validated methods of automated comparison and clustering will greatly expand the scope of molecular epidemiology.

The combination of conventional epidemiologic investigations with molecular techniques for genotyping pathogens has elucidated the epidemiology of many infectious diseases. The most frequently used genotyping techniques (e.g., pulsed-field gel electrophoresis, restriction fragment length polymorphism [RFLP], and randomly amplified polymorphic DNA) yield fragment-based data. Fewer than 100 patterns can be compared visually. For larger numbers, commercially available computer programs can be used to identify a manageable subset of potentially matching patterns, which are then compared visually. This approach is accurate but cumbersome and excessively labor-intensive as the number of isolates exceeds several hundred. Furthermore, the results of computer-assisted matching are not as reproducible as systematic computational methods. These limitations significantly constrain the size, scope, and standardization of molecular epidemiologic investigations. We present an approach by which identical patterns can be identified from large collections of DNA fingerprints.

The number of IS*6110* fingerprints continues to increase, with many studies across the globe producing IS*6110* data to characterize *Mycobacterium tuberculosis* isolates. Such molecular epidemiologic studies provide information about tuberculosis (TB) transmission patterns. Studies in Ethiopia, Tunisia, and The Netherlands (1), South Africa (2), India (3), Denmark and Greenland (4,5), the United States (6), and Tanzania (7), among many others, exploit IS*6110*-based RFLP genetic fingerprints.

We developed an automated computational system, in which a statistical analysis of the error in measuring fragment sizes provides a conceptual framework for comparing sets of fragment lengths. The computational approach to lane comparison—align-and-count method (ACM)—permits calculation of the number of fragments that match between two IS*6110*-based RFLP fingerprints. The parameters of the computational ACM are adjusted to provide the same high

Address for correspondence: Hugh Salamon, Division of Infectious Diseases and Geographic Medicine, Stanford University Medical Center, Rm S 156, Stanford, CA 94305-5107, USA; fax: 650-498-7011; e-mail: hugh@molepi.stanford.edu.

sensitivity as the labor-intensive visual inspection used over the last 5 years.

We developed an approach to identifying a set of identical fingerprints when the identity of the fingerprints is nontransitive. We also explored improving the specificity among matched fingerprints to reveal additional information in RFLP lanes.

### Data Acquisition

Genotyping *M. tuberculosis* isolates with IS*6100*-based RFLP fingerprinting was performed as described in van Embden et al., 1993 (8). Computer-assisted comparison and clustering were performed on the RFLP lanes as described in Woelffer et al., 1996 (9).

Internal standards were used to quantitate fragment sizes visualized with a DNA probe to IS*6110*. Two films' exposures were scanned into Whole Band Analyzer software (BioImage, Ann Arbor, MI, USA); one was obtained when probing for the internal standard, the other when probing for IS*6110*. The resulting images were aligned with three registration marks that gave reference to the original nylon membrane. The Whole Band Analyzer software quantitated fragment lengths for the IS*6110*-visualized bands, which were inspected and edited manually in the software package. The resulting collections of fragment lengths for each lane (bacterial isolate or laboratory strain H37Rv) were exported to our ACM software and compared with other lanes.

### Mathematical Methods

The following is a descriptive summary of the principles underlying the analysis (Appendix).

### Analysis of Error in Data

The magnitude and characteristics of experimental error were empirically assessed by analyzing variation in the results obtained from a reference strain included in each experiment (gel). The absolute and proportional differences in the measured fragment lengths of biologically identical samples of this strain were calculated; results showed that the error in measurement was proportional to fragment length and greater between than within gels.

### Align-and-Count Matching Algorithm

A method for matching was developed to accommodate the empirically defined error. The fragment length data from two lanes were scaled through a range of values, and the maximum number of mutually closest bands falling within a threshold tolerance was reported (Figure 1). The acceptable tolerance was smaller when lanes from the same gel were compared than when lanes from different gels were compared. An animated demonstration of this method is accessible on the Internet (URL for use with a graphics-capable Web browser: http://molepi.stanford.edu/hugh/acm/counting).

### A Graph-Theoretic Approach to Identical Fingerprints

We considered pairs of patterns identical if all fragments matched. However, because the results might be nontransitive (A might be identical to B, B identical to C, but A not identical to C), the identification of groups of matched patterns is more complicated. A graph-theoretic approach was used to assemble clusters of matching fingerprints.

### Alignment and Analysis of Residual Error

We further aligned collections of lanes determined to match according to the above algorithms. The optimal alignment was defined as that which minimized the proportional error between putatively identical fragments. This analytic step, which is comparable to the experimental approach of rerunning clustered strains in the same gel, improved the ability to distinguish similar, but nonidentical fingerprints.

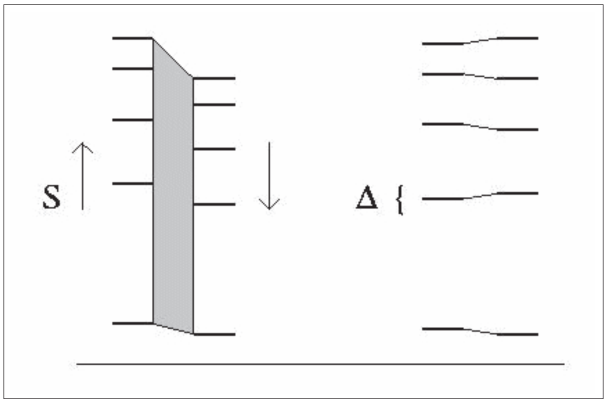

Figure 1. The align-and-count method finds the maximum number of mutually closest bands within a threshold deviation value **Δ**, for a search across a range *S* of scaling values. The two lanes are scaled incrementally, thus searching for the best alignment.

### Error in Analysis of H37Rv Data

Investigating the absolute error for pairs of 116 H37Rv lanes (Figure 2), we found that the error was consistently higher in gel-to-gel comparisons than in comparisons of lanes from the same gel. Error was proportional to fragment length for the range of fragment lengths from 0.9 kbp up to at least 5 kbp (Figure 2), which included 90% of the bands in the fingerprint data from San Francisco. The latter empirical result was consistent with the fact that the distance migrated by a DNA fragment on an electrophoresis gel is typically proportional to the logarithm of the fragment length. Furthermore, the error found when we compared one band of a lane to that band in another lane was positively correlated to the error found for the other bands; in other words, if the first band in lane A was larger than the average measurement for that band, it was likely that the other bands in lane A would be larger than the average measurements. This positive correlation was intuitively evident in comparing lane maps (i.e., when comparing graphic representations of the fragment lengths). In Figure 3, the set of lane maps on the left are measurements of a genotype found in San Francisco. With the exception of the fourth lane from the right, they represent a set of identical patterns. Note that the error is mostly a scaling error and that if one fragment is larger than average for that lane, the others are very likely to be larger also. These two observations—that error is proportional to fragment length and positively correlated for bands within a given lane—suggests two classes of error: one is a property of each band; the second is a property of each lane. This analysis motivated us to develop a computational algorithm that scales fingerprints and measures the number of mutually closest bands within threshold sizes of each other for the best alignment, i.e., the scaling that maximizes the number of matching fragments.

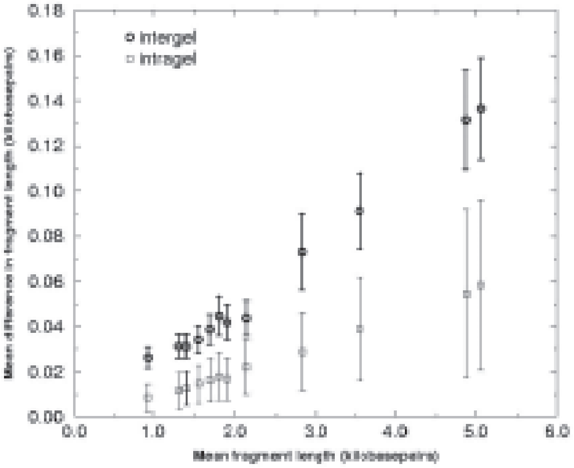

Figure 2. Means and two standard errors of the mean error bars for pairwise comparisons among 116 12-banded H37Rv lanes show that error is consistently larger when comparing lanes between gels than when comparing lanes from the same gel. The x-axis corresponds to *w(b)*, and the y-axis to *d(b)*, as presented in the text. It is evident that error is proportional to fragment length in the range of fragment lengths found in H37Rv. The data exhibit 2% to 3% error for between gel comparisons, but only approximately 1% error on average for within gel comparisons.

### Alignment and Residual Error for H37Rv Lanes

By optimally aligning (i.e., minimizing the sum of proportional errors) pairs of H37Rv lanes, we find a distribution of scaling factors and of residual error. Table 1 shows 6,641 pairwise comparisons of 91 replicate lanes (members of each pair are taken from different gels). The mean value of *s*, the scaling factor, for these 6,641 alignments is 0.0212, and the standard deviation of *s* is 0.0189. The reduction in error due to

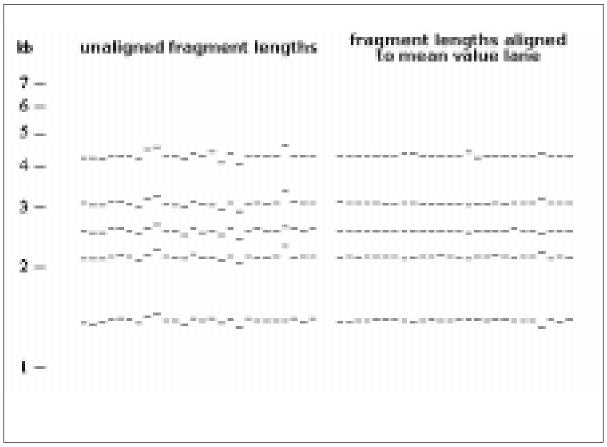

Figure 3. Additional alignment of very similar patterns can identify clearly distinct patterns. Measurement noise obscures the detailed relationships between 26 patterns that were identified from 1,335 as being very similar. However, after alignment to a consensus pattern, a clearly distinct pattern (an outlier from the other members of this autocluster) can be readily identified. Fragment lengths are given in kilobasepairs (kb).

Table 1. Pairwise comparisons (n=6,641) of lanes across gels characterize unaligned proportional error and residual error

| H37Rv band $b$* | Mean kilo-bases $\bar{w}_b$ | Unaligned pairwise proportional error mean $\bar{r}(b)$ | s.d. | Aligned pairwise proportional error mean $\bar{r}^a(b)$ | s.d. |
|---|---|---|---|---|---|
| 1 | 5.029 | 0.0253 | 0.0196 | 0.0099 | 0.0079 |
| 2 | 4.853 | 0.0249 | 0.0202 | 0.0084 | 0.0075 |
| 3 | 3.533 | 0.0253 | 0.0303 | 0.0109 | 0.0203 |
| 4 | 2.814 | 0.0237 | 0.0283 | 0.0105 | 0.0182 |
| 5 | 2.153 | 0.0202 | 0.0186 | 0.0086 | 0.0125 |
| 6 | 1.892 | 0.0210 | 0.0204 | 0.0100 | 0.0085 |
| 7 | 1.800 | 0.0226 | 0.0220 | 0.0092 | 0.0082 |
| 8 | 1.684 | 0.0227 | 0.0193 | 0.0083 | 0.0083 |
| 9 | 1.541 | 0.0221 | 0.0198 | 0.0079 | 0.0081 |
| 10 | 1.397 | 0.0225 | 0.0200 | 0.0093 | 0.0078 |
| 11 | 1.314 | 0.0231 | 0.0200 | 0.0111 | 0.0090 |
| 12 | 0.0936 | 0.0281 | 0.0249 | 0.0142 | 0.0157 |
| mean | | 0.0235 | 0.0219 | 0.0099 | 0.0110 |

*Symbols as in Appendix.

alignment is approximately twofold, to approximately 1% with a standard deviation also of approximately 1% (Table 1); therefore, a search range, $S$, of 0.10 will allow for virtually every incidence of scaling error in the data. (Assuming normally distributed scaling and noting that 0.10 lies more than four standard deviations about the mean scaling error, we conclude that scaling error will not be compensated in fewer than 1 out of 10,000 independent pairwise comparisons.) Employing a deviation tolerance, $\Delta$, of 0.045 should ensure a sensitivity very close to 100% for matching individual bands, after alignment. (Assuming normally distributed differences between replicate band measurements and a deviation tolerance of 4.5%, which is approximately 3.5 standard deviations above the mean fragment length error after alignment, one should falsely conclude two identical bands do not match with an approximate probability of 0.0002.) These parameter values, together with the number of incremental searches, $I$, set to 100, empirically gave results agreeing closely with visual inspection by experienced researchers who matched entire lanes. Similarly, one may use analysis of within gel lane error to determine the parameter values for the ACM to match lanes from the same gel.

Both the range of scaling factors and the threshold deviations are derived from empirical investigation of the San Francisco data. An adjustment for larger error in measurement is included for the more rare larger fragment length bands. In applying the ACM to San Francisco bacterial fingerprints, $\Delta$ is allowed to increase at a rate of 0.005/kbp above a value of 7 kbp.

### San Francisco Bacterial Genetic Fingerprint Comparisons

To evaluate the performance of the ACM, we analyzed (by computer-assisted visual inspection and by ACM) 125 lanes from bacterial isolates obtained in the first half of 1996. We evaluated ACM's performance by first determining whether a 1996 lane matched all bands to lanes in visually defined clusters (from previous years), matched other 1996 lanes, or did not find any identical matches at all. The automated matching of the 1996 lanes agreed nearly perfectly with the visual analysis; the few conflicting results were due to inconsistencies in the existing data (e.g., two bands of nearly identical size being edited sometimes as one band and sometimes as two). Humans often compensate for such inconsistencies, whereas a computational method would have to have such capabilities explicitly built in.

Using ACM, we analyzed all 890,445 pairwise comparisons of isolates from 1,335 TB cases in San Francisco from 1991 to mid-1996. The autoclusters defined from the pairwise comparisons agreed closely with the clusters defined by computer-assisted visual inspection (Table 2). An example of one autocluster is shown in Figure 3. Without additional alignment, noise in fragment length measurements makes it difficult to determine if these putative clusters include individual patterns which, although similar, are distinct (outliers) or if the cluster contains identifiable subgroups of patterns (subclusters).

We then further analyzed autoclusters and identified more precisely nonidentical genotypes. Refinement of clusters begins with defining a consensus pattern for the cluster (consisting of the collection of mean fragment lengths for each band). Then the fragment lengths for each putative member of the autocluster are aligned to

Table 2. Preliminary autoclustering agrees closely with results obtained by computer-assisted visual inspection (CAVI)

| | Clustered by CAVI | Not clustered by CAVI | Total |
|---|---|---|---|
| Autoclustered | 540 | 27 | 567 |
| Not autoclustered | 49 | 719 | 768 |
| Total | 589 | 746 | 1,335 |

the consensus pattern. After alignment, a pattern can be easily identified as an outlier (Figure 3).

If the analysis of a refined, aligned cluster shows multiple outliers, realignment of subsets of lanes can be used to reveal subclusters of identical patterns. In Figure 4 the distributions of fragment lengths before (a,b) and after (c,d) an initial alignment are presented for an autocluster of 84 2-banders from San Francisco. Given that the aligned bands are clearly split into two distributions, we split the autocluster into two subclusters. A set of 26 fingerprints (group 1) is aligned to its assembled mean-value lane (Figure 4 e,f), as is a set of 58 fingerprints (group 2, Figure 4 g,h). The contrast between the original fragment length data and the two well-aligned groups of fingerprints shows that the higher fragment length band is shifted between the two groups and no clear outliers exist after alignment. Figure 5 shows that alignment greatly improves a difficult-to-resolve clustering issue among four lanes.

Preliminary investigation with a polymorphic GC-rich sequence (PGRS) fingerprinting method shows that the two subclusters exhibit distinct fingerprints, which further validates the increased specificity in IS*6110* fingerprints. Of 81 PGRS genotyped autoclustered 2-banders, 63 fall into eight visually defined clusters, the remaining being unique PGRS patterns among the members of the IS*6110* 2-banded autocluster. Each cluster consists of isolates that all fall into one or the other IS*6110*-refined subcluster.

### Conclusions

We have developed and validated a systematic approach to pairwise comparison and clustering of identical patterns in a large data set of DNA fragment-based genotypes. Incorporating a control pattern in each experiment allows the nature and magnitude of error in DNA fragment length measurements to be determined. An analysis of measurement error provides parameter values to use with algorithms that accommodate these errors. Relative scaling of entire lanes, an important characteristic of the error generated in quantitating fragment lengths from RFLP patterns used to type *M. tuberculosis* isolates, arises in part from aligning two images of a gel, one for internal lane size standards and one for data fragments; the image of internal standards and the image of data fragments are registered by three marks. Error in registration of the two images occurs, leading to the scaling effect in the fragment lengths reported. We strongly suggest that software for analyzing internal lane size standards and data fragments from separate images permit (and encourage) use of more than three registration marks. While internal standards compensate for idiosyncrasies in lane-specific fragment mobility, the limitations imposed by poor registration methods can result in increased scaling error. We have demonstrated that allowing for scaling, as in ACM, greatly assists in automating matching; incorporating alignment of pairs of lanes into the method has provided fully automated lane matching that agrees closely with results of the well-established method of computer-assisted visual comparison. We successfully address the nontransitivity of pairwise identity and once again use scaling of lanes to ensure the reliability of automated clustering of identical patterns.

Alignment of DNA fragment patterns removes noise in clusters of fingerprints, showing further specificity in genotyping. This is analogous to the experimental approach of rerunning similar patterns on a single gel to reduce intergel noise. Mathematical transformation of the fragment length data yields similar information for far less cost in labor and materials. Further automated clustering within sets of aligned patterns could exploit the fact that we assume putative homology of fragments. As the aligned patterns have the same number of fragments, the residual error presents a multidimensional clustering problem; each pattern may deviate from the mean-value pattern in any of the fragment lengths. This clustering may prove more straightforward than the more general problem of clustering among patterns differing in numbers of fragments.

Numerous commercial packages of computer software are available to compare and match DNA fragment patterns. The availability of these systems fosters unquestioning application of turnkey pattern matching and clustering methods, which are not always fully documented or validated (for fragment-based genotypes) in the scientific literature. While possibly acceptable for small studies when visually validated, this approach to data analysis is risky for large studies. The methods for matching DNA fragment patterns presented in this paper should be an adjunct to software packages that quantitate fragments. We provide a systematic approach to analysis of fragment length

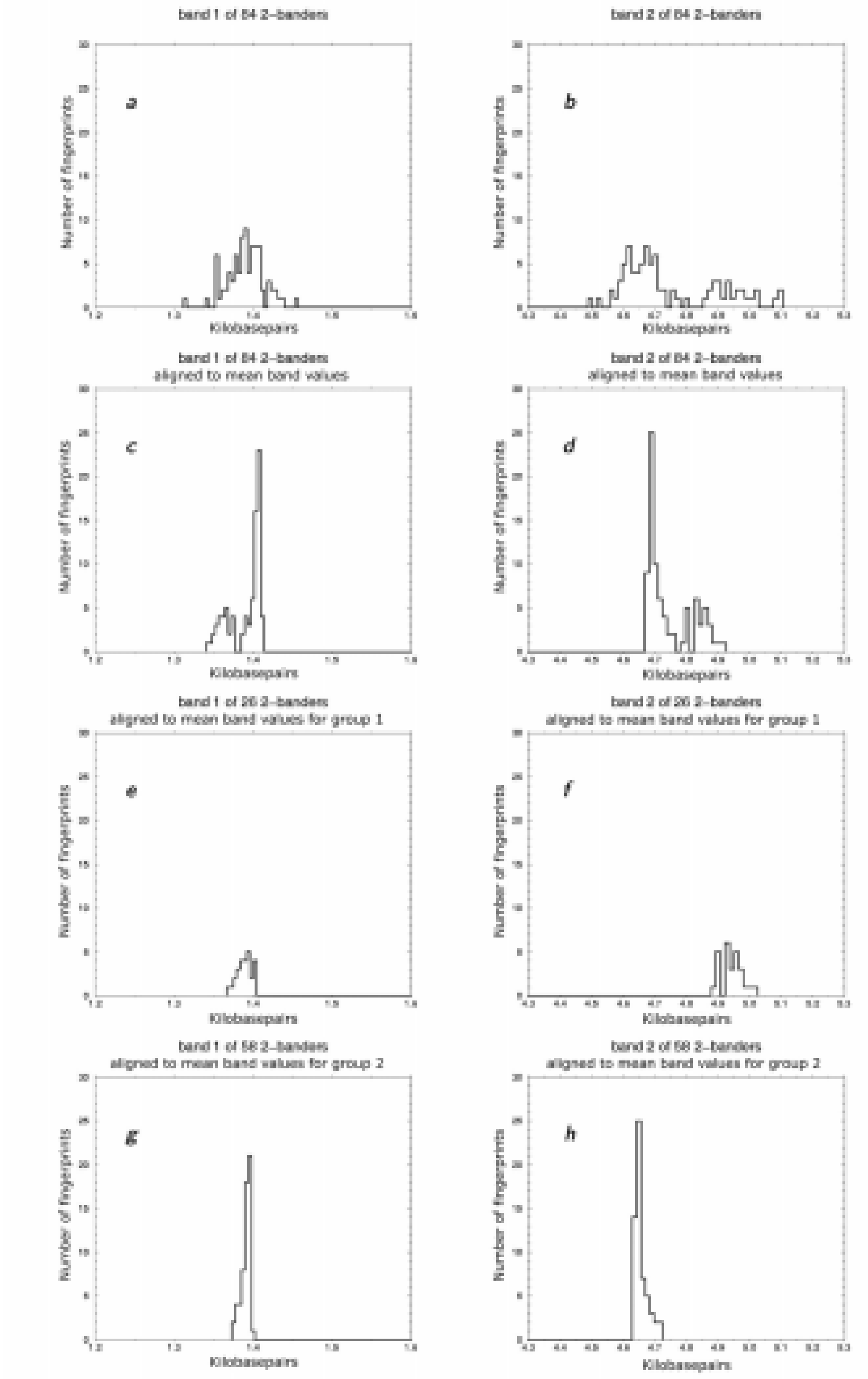

Figure 4. Histograms of the fragment lengths for 84 two-banded patterns connected by identity (autoclustered with in-house software) exhibit enough spread in values to make detecting outliers and band shifts difficult (a,b). Aligning the 84 lanes to the mean-value lane for this collection reveals that the lanes do not align well, but instead shows bimodal distributions for the fragment lengths (c,d). Dividing the 84 fingerprints into two sets and separating the distinct distributions detected when aligning all 84 fingerprints show that 26 fingerprints align well to their mean-value lane (e,f), and the remaining 58 also align well to their respective mean value lane (g,h). The smaller fragment length fragment does not appear shifted between the two sets of 2-banders (comparing e to g), but the larger fragment is clearly shifted (comparing f to h).

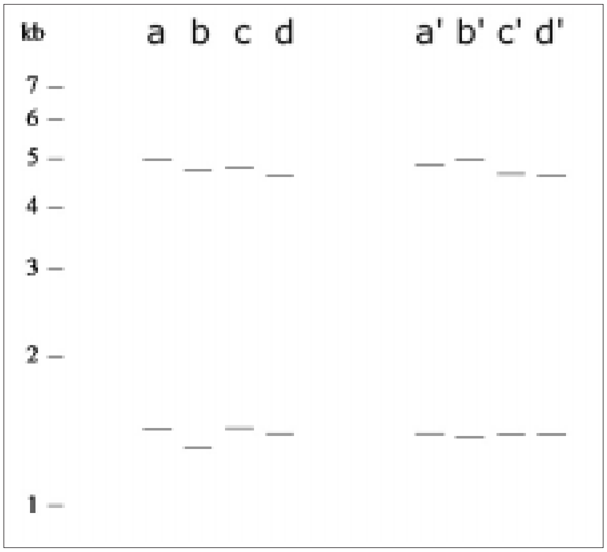

Figure 5. Prior to alignment of two sets of 2-banders, lanes are difficult to cluster (lanes a-d are from the distributions in Figures 4a and 4b). Subsequent to alignment, lanes are much easier to cluster (lanes a′ and b′ are specific examples from the distributions in Figures 4e and 4f; lanes c′ and d′ likewise correspond to Figures 4g and 4h). Fragment lengths are given in kilobasepairs (kb).

estimates, appropriate whether the numerical data are generated by hand using a ruler and arithmetic or are output by a multithousand dollar gel analyzer. This approach can thus be used by molecular epidemiologists working with both large and small budgets around the world. We anticipate that our methods can be incorporated into existing commercial software packages with broad distribution and encourage similar documentation in peer-reviewed journals of other methods provided in software packages.

In addition, our focus on the use of fragment length data, as opposed to the comparison of actual images, will foster comparison of data generated in different laboratories that use different proprietary software. We are working on a World-Wide Web–based system to facilitate IS*6110* genotype data sharing.

The availability of a precise and validated method to count the number of matching fragments in a pairwise fashion among very large numbers of patterns now permits an assessment of the importance and usefulness of approaches that exploit fingerprint similarity. In conjunction with the growing understanding of the underlying biology of IS*6110* instability and the relevant statistical issues, this may greatly expand the scope of TB molecular epidemiology.

A general question arises when comparing molecular fingerprints: how many bands need to match to indicate a close biologic relationship? Aside from the issues of defining biologic “closeness”—too often ignored in epidemiologic studies—technical issues are relevant to ACM. The usefulness of the matched band information output by ACM depends on at least several factors: the underlying band size distribution from which fingerprint bands are sampled, the independence of sampled bands, measurement error (both scaling and independent band error), the length of the gel, and the number of fragments. At one extreme, where error is large and few bands are observed, even in the case of a perfect match, statistical analysis may fail to reject coincidental band matches. By using computer simulation and sets of assumptions regarding band size distributions, one may learn about the role of coincidental band matching. We are actively researching these issues for IS*6110* fingerprint comparisons. Furthermore, we intend to provide a general computational framework in which one may assess error in laboratory measurement, the appropriateness of ACM for analyzing data (and appropriate parameter values to use with the method), and the role of coincidence in band matching. Information regarding computer programs for various tasks, including ACM matching itself, will be made available on the Internet at http://molepi.stanford.edu/hugh/acm.

A study of *M. tuberculosis* isolates from northern Tanzania demonstrates the utility of partial matching (3). The study brings into focus difficulties inherent in employing one-parameter tolerance for DNA fragment-based genotype matching, a technical issue effectively addressed by ACM. Gillespie et al. (7) also call attention to the poor specificity of low copy number IS*6110*-based fingerprints, exacerbated by the use of the Dice coefficient. We are pursuing alternative similarity measures that use the numbers of matching fragments identified by ACM and are tailored to the needs of epidemiologic investigations. Dendogram clustering methods often provided in software targeted at DNA-fragment genotype management and analysis could in some instances fail to reconstruct the correct relationships among infectious organism isolates, even when presented perfect clock-like genetic distances. This may result in part from the fact that fast-evolving markers are characterized for isolates sampled over a period; samples are not contemporaneous. In conjunction with our efforts to define similarity measures, we are also working to modify phylogenetic inference tools used in clinical and molecular epidemiologic settings to better handle the data typically analyzed in molecular fingerprint management and analysis software.

### Appendix

### Analysis of Error in Data

To characterize error in fragment length measurement, pairs of 12 band 7H37Rv lanes are compared; the difference between fragment lengths of each band is calculated as follows. Let $w_{i,b}$ be the measured fragment length of band $b$ of lane $i$. In general, let $B$ be the number of bands. To compare lanes $i$ and $j$, we can calculate the absolute difference between the measured lengths,

$$d_{i,j}(b) = |w_{i,b} - w_{j,b}|.$$

Let $\delta_{i,j}$ be an indicator that equals 1 when lanes $i$ and $j$ are from the same electrophoresis gel and 0 otherwise. Let $n$ be the total number of replicate lanes. The mean absolute difference for a fragment over all pairwise comparisons of lanes from different gels is found by,

$$\overline{d(b)} = \frac{1}{t}\sum_{i=1}^{i=n-1}\sum_{j>i}^{j=n} d_{i,j}(b)\cdot(1-\delta_{i,j}),$$

where $t = \sum_{i=1}^{i=n-1}\sum_{j>i}^{j=n}(1-\delta_{i,j})$ is the number of pairs of lanes from different gels.

Similarly, we calculate the proportional difference between measurements for a fragment,

$$r_{i,j}(b) = \frac{|w_{i,b} - w_{j,b}|}{(w_{i,b} + w_{j,b})/2},$$

and its mean for comparisons between lanes from different gels,

$$\overline{r(b)} = \frac{1}{t}\sum_{i=1}^{i=n-1}\sum_{j>i}^{j=n} r_{i,j}(b)\cdot(1-\delta_{i,j}).$$

Calculating fragment measurement errors for the $u = \sum_{i=1}^{i=n-1}\sum_{j>i}^{j=n}\delta_{i,j}$ pairwise comparisons of lanes from the same gel are also performed.

### Align-and-Count Matching Algorithm

The Align-and-Count Method (ACM) for counting the matching bands between two lanes is defined as follows. Consider two lanes, lane $A$ with $m$ bands,

$$w_{A,x},\ x = 1,...,m,$$

and lane $B$ with $n$ bands,

$$w_{B,y},\ y = 1,...,n.$$

We count the mutually closest measured fragment lengths within a proportional deviation factor, $\Delta$, over a range of alignments (Figure 1). Alignments are searched by scaling the fragment lengths. Multiplying the fragment lengths in a lane by a scaling factor reflects the phenomenon that error in fragment lengths is proportional to fragment length and is positively correlated for fragments in a lane.

Define

$$\text{Match }(w_{A,x}, w_{B,y}) = \begin{cases} 1 & \text{if } |w_{B,y} - w_{A,x}| \le \Delta \text{ and } x \text{ and } y \text{ are mutually closest bands} \\ 0 & \text{otherwise} \end{cases}$$

By mutually closest we mean that there is no band in lane $B$ closer to band $x$ of lane $A$ than band $y$, and there is no band in lane $A$ closer to band $y$ of lane $B$ than band $x$.

Lanes are incrementally scaled ($I$ increments), and the maximum number of matching bands is reported. Specifically, the $J^{th}$ increment in the search scales the lanes as follows:

$$\Omega_{A,x}(J) = w_{A,x} * (1 + (J - I/2) * S), \quad x = 1, \ldots, m,$$

and

$$\Omega_{B,y}(J) = w_{B,y} * (1 + (I/2 - J) * S), \quad y = 1, \ldots, n.$$

Here $S$ defines the range of scaling factors (Figure 1). In this way the lanes slide past each other as $J$ goes from 1 to $I$, always scaling the bands proportionally. At one extreme, lane $A$ is scaled ($S$/2 * 100)% larger while $B$ is scaled ($S$/2 * 100)% smaller. At the other extreme the scaling is reversed.

The number of matching bands reported, k, is the maximum of K($J$) over $J$ = 1,...,$I$:

$$K(J) = \sum_{x=1}^{x=m} \sum_{y=1}^{y=n} \text{Match}(\Omega_{A,x}(J), \Omega_{B,y}(J)).$$

We have described a method to find the number of matching bands $k$, when comparing a lane with $m$ bands and a lane with $n$ bands. This algorithm has three parameters: $S$, the range of scaling factors to align the lanes, $\Delta$, the cutoff proportional difference for mutually closest bands to be considered matches, and $I$, the number of increments used to search the range of scaling. Note that $\Delta$, in general, may be a function of the fragment lengths of mutually closest bands under inspection.

### A Graph-Theoretic Approach to Identical Fingerprints

If all bands in lane $j$ match their aligned counterparts in lane $i$, the two lanes are defined as identical. A common feature of ACM and all band-sharing approaches is that identity may not be transitive. This situation may arise when accumulated errors exceed tolerance or (proportionally) small changes in fragment size occur in biologic samples.

To analyze identical fingerprints, we define sets of fingerprints connected by identity. Let each of T fingerprints exhibiting the same number of bands be a node in a graph; let an edge joining two nodes indicate that two fingerprints are identical. By an algorithm attributable to Dijkstra (10), we determine the number of steps, P, of identity between two fingerprints when such a pathway exists. The algorithm finds the shortest path between two nodes. We use a code modified from that presented in Tenenbaum et al. (10). From this analysis we assemble collections of fingerprints connected by identity, which serve as tentative clusters of identical fingerprints. We refer to these tentative clusters as autoclusters. The distributions of fragment lengths for these collections of lanes are subsequently plotted, allowing outliers and band-shifts to be identified.

### Alignment and Analysis of Residual Error

We aligned two replicate fingerprint patterns by using the following least squares regression formula. We minimize

$$Q(s) = \sum_{b=1}^{B} \left(\log(w_{i,b}) - \log(s \cdot w_{j,b})\right)^2$$

with respect to the scaling factor s by which lane $j$ is aligned to lane $i$. Log-transformed fragment lengths are used to reflect the fact that measurement error is proportional to fragment length. The minimization has the closed form solution

$$s = \exp\left(1/B \sum_{b=1}^{B} \log(\frac{w_{i,b}}{w_{j,b}})\right).$$

Residual error in band size measurement after lane alignment is evaluated in a fashion analogous to that used for unaligned lanes. For example, the proportional difference between band $b$ of lane $j$ aligned to lane $i$ and band $b$ of lane $i$ is

$$r_{i,j}^{a}(b) = \frac{|w_{i,b} - s \cdot w_{j,b}|}{(w_{i,b} + s \cdot w_{j,b})/2}.$$

For alignment of sets of lanes, the mean fragment length is calculated for each fragment. A mean-value lane is constructed from these mean lengths. Each lane is then aligned to the mean-value lane. The alignment is plotted and analyzed for outliers, providing refined clusters of identical fingerprints.

### Acknowledgments

We thank Philip Hopewell, for his interest in and support for continued development of *M. tuberculosis* genetic fingerprinting in TB research; Mike Cummings, for his participation in numerous meetings regarding analysis of the fingerprint data, his input and discussions in the evolution of ideas leading to the work presented herein, and his comments on manuscripts; Melvin Javonillo, for help in accessing fingerprint data; Daniel Ruderman, for help with the issue of logarithmic alignment; and Marcel Behr and Sam Warren, for comments on manuscripts.

This research was supported by NIH grants AI35969, AI34238.

Hugh Salamon is a postdoctoral researcher at Stanford University Medical Center; he is focusing on molecular epidemiology and *Mycobacterium tuberculosis* genomics. In his work as computer programmer in the Department of Medicine at the University of California, San Francisco, he writes custom software for many analyses and maintains a web site (http://molepi.stanford.edu) at Stanford University.

### References

1. Hermans PW, Messadi F, Guebrexabher H, van Soolingen D, de Haas PE, Heersma H. et al. Analysis of the population structure of *Mycobacterium tuberculosis* in Ethiopia, Tunisia, and the Netherlands: usefulness of DNA typing for global tuberculosis epidemiology. J Infect Dis 1995;171:1504-13.
2. Warren R, Hauman J, Beyers JN, Richardson M, Schaaf HS, Donald P, van Helden P. Unexpectedly high strain diversity of *Mycobacterium tuberculosis* in a high-incidence community. S Afr Med J 1996;86:45-9.
3. Das S, Paramasivan CN, Lowrie DB, Prabhakar R, Narayanan PR. IS*6110* restriction fragment length polymorphism typing of clinical isolates of *Mycobacterium tuberculosis* from patients with pulmonary tuberculosis in Madras, South India. Tubercle and Lung Disease 1995;76:550-4.
4. Yang ZH, de Haas PE, Wachmann CH, van Soolingen D, van Embden JD, Andersen AB. Molecular epidemiology of tuberculosis in Denmark in 1992. J Clin Microbiol 1995;33:2077-81.
5. Yang ZH, de Haas PE, van Soolingen D, van Embden JD, Andersen AB. Restriction fragment length polymorphism *Mycobacterium tuberculosis* strains isolated from Greenland during 1992: evidence of tuberculosis transmission between Greenland and Denmark. J Clin Microbiol 1994;32:3018-25.
6. Barnes PF, el-Hajj H, Preston-Martin S, Cave MD, Jones BE, Otaya M, et al. Transmission of tuberculosis among the urban homeless. JAMA 1996;275:305-7.
7. Gillespie SH, Kennedy N, Ngowi FI, Fomukong NG, al-Maamary S, Dale JW. Restriction fragment length polymorphism analysis of *Mycobacterium tuberculosis* isolated from patients with pulmonary tuberculosis in northern Tanzania. Trans R Soc Trop Med Hyg 1995;89:335-8.
8. van Embden JD, Cave MD, Crawford JT, Dale JW, Eisenach KD, Gicquel B, et al. Strain identification of *Mycobacterium tuberculosis* by DNA fingerprinting: recommendations for a standardized methodology. J Clin Microbiol 1993;31:406-9.
9. Woelffer GB, Bradford WZ, Paz A, Small PM. A computer-assisted molecular epidemiologic approach to confronting the reemergence of tuberculosis. Am J Med Sci 1996;311:17-22.
10. Tenenbaum AM, Langsam Y, Augenstein MJ. Data structures using C. Englewood Cliffs (NJ): Prentice-Hall; 1990. p. 514-6.
